# P-1378. Thrombocytopenia and Risk Factors Associated with Linezolid and Contezolid Treatment in Pulmonary Tuberculosis Patients

**DOI:** 10.1093/ofid/ofaf695.1565

**Published:** 2026-01-11

**Authors:** Li Wen, Yan Liu, Xuan Lei

**Affiliations:** Beijing Chest Hospital, Capital Medical University, Beijing Tuberculosis & Thoracic Tumor Research Institute, Beijing, Beijing, China (People's Republic); Beijing Chest Hospital, Capital Medical University, Beijing Tuberculosis & Thoracic Tumor Research Institute, Beijing, Beijing, China (People's Republic); Beijing Chest Hospital, Capital Medical University, Beijing Tuberculosis & Thoracic Tumor Research Institute, Beijing, Beijing, China (People's Republic)

## Abstract

**Background:**

This retrospective cohort study aims to comparatively analyze the incidence and risk factors of thrombocytopenia associated with linezolid and contezolid treatment in pulmonary tuberculosis patients.

**Methods:**

Pulmonary tuberculosis patients who received linezolid or contezolid for anti-tuberculosis treatment for at least 7 days between 2019 and 2023 were selected. Platelet levels were monitored during the first three months of treatment. The incidence, onset time, and risk factors of thrombocytopenia associated with both drugs were evaluated.

**Results:**

A total of 120 pulmonary tuberculosis patients treated with linezolid and 90 patients treated with contezolid were included. The mean treatment duration was (62.5±15.3) days and (58.8±12.7) days respectively (P=0.228). All patients completed at least 90 days of clinical endpoint observation, with a median follow-up time of 123 days (IQR 98-158). Patient baseline characteristics are shown in Table 1.

The incidence of thrombocytopenia in the linezolid group was 37.5% (32 cases with oral administration [71.1%], 13 cases with intravenous infusion [28.9%]), significantly higher than the 8.89% (8/90) in the contezolid group (*P* < 0.001). The median time to thrombocytopenia was day 10 after treatment initiation in 32 patients of the linezolid group, compared to day 58 in 8 patients of the contezolid group, with a statistically significant difference (P < 0.001). The Kaplan-Meier curve (see Figure 1) demonstrated that as the observation period extended, the cumulative incidence of thrombocytopenia in the linezolid group increased significantly compared to the contezolid group.

Multivariate logistic regression analysis identified independent risk factors for thrombocytopenia in the linezolid group, including decreased baseline platelet count (OR=0.98, P=0.021), advanced age (OR=2.15, P=0.012), and renal dysfunction (OR=1.93, P=0.037).

**Conclusion:**

The risk of thrombocytopenia associated with linezolid treatment for pulmonary tuberculosis was significantly higher than with contezolid, with an earlier onset time. Decreased baseline platelet count, advanced age, and renal dysfunction were identified as independent risk factors for linezolid-induced thrombocytopenia.

**Disclosures:**

All Authors: No reported disclosures

